# Chemistry and Photochemistry of Anthocyanins and Related Compounds: A Thermodynamic and Kinetic Approach

**DOI:** 10.3390/molecules21111502

**Published:** 2016-11-10

**Authors:** Nuno Basílio, Fernando Pina

**Affiliations:** LAQV, REQUIMTE, Departamento de Química, Faculdade de Ciências e Tecnologia, Universidade Nova de Lisboa, 2829-516 Caparica, Portugal

**Keywords:** anthocyanins, flavylium compounds, energy level diagram, multistate chemistry, cyclodextrins, cucurbiturils

## Abstract

Anthocyanins are identified by the respective flavylium cation, which is only one species of a multistate of different molecules reversibly interconverted by external inputs such as pH, light and temperature. The flavylium cation (acidic form) is involved in an apparent acid-base reaction, where the basic species is the sum of quinoidal base, hemiketal and *cis*- and *trans*-chalcones, their relative fraction depending on the substitution pattern of the flavylium cation. The full comprehension of this complex system requires a thermodynamic and kinetic approach. The first consists in drawing an energy level diagram where the relative positions of the different species are represented as a function of pH. On the other hand, the kinetic approach allows measuring the rates of the reactions that interconnect reversibly the multistate species. The kinetics is greatly dependent on the existence or not of a high *cis*-*trans* isomerization barrier. In this work, the procedure to obtain the energy level diagram and the rates of inter-conversion in the multistate in both cases (low or high isomerization barrier) are described. Practical examples of this approach are presented to illustrate the theory, and recently reported applications based on host–guest complexes are reviewed.

## 1. Introduction

### 1.1. The Flavylium-Based Multistate—A Brief History

The comprehension of the reversible multistate of chemical reactions involving 2-phenyl benzopyrylium (flavylium cation) is a result of more than one century of research, since the first synthesis of 4-methyl-7-hydroxyflavylium prepared by Büllow and Wagner [1]. The most significant flavylium-based multistates are anthocyanins. These natural compounds are generally extracted or synthesized in acidic medium in the form of the red flavylium cation. Immediately after dissolution of an anthocyanin (in the flavylium cation form) in water, a blue colour appears, but disappears relatively fast to give a colourless solution. After the disappearance of the blue colour (or before), the addition of some drops of concentrated acid restores the original red colour of the flavylium cation. In order to explain this peculiar behaviour, many decades of research and the work of a large number of scientists [2,3] including two Nobel prizes, Richard Willstätter 1915 [4], and Richard Robinson, 1947, were necessary [5].

**Scheme 1:**
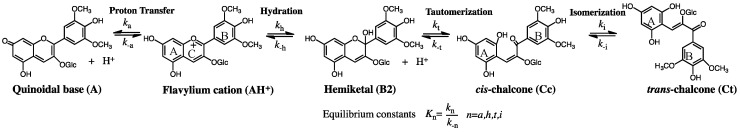
Multistate of oenin in acid to moderately acid solutions.

Scheme 1 summarizes the four reversible chemical reactions that occur in anthocyanins and related compounds, in a pH range from 1 to moderately acidic, exemplified for malvidin-3-glucoside (oenin) [6].

Five different chemical species reversibly interconnected by four different chemical reactions can be observed defining a multistate. Inspection of Scheme 1 shows that, according to Chatelier’s principle, at lower pH values the multi-equilibrium is shifted towards the flavylium cation. This generally occurs in very acidic solutions, for example at pH ≤ 1 for anthocyanins. The multistate is conveniently studied by means of direct pH jumps defined by the addition of base to equilibrated solutions of flavylium cation. Reverse pH jumps should also be used and, in this case, the solutions are previously equilibrated at higher pH values and acid is added. A direct pH jump triggers a succession of reactions as reported in Scheme 1. The proton transfer that converts flavylium cation (**AH^+^**) in quinoidal base (**A**) occurs on a timescale of micro-seconds. Proton transfer can be followed using temperature jumps [7] or, in some favourable cases, flash photolysis, [8] but is too fast for the stopped flow equipment. The species **AH^+^** and **A** are in fast equilibrium and behave as a single one in the subsequent kinetic processes (the fraction **AH^+^** and **A** is dependent on the respective p*K*_a_ and pH of the solution). In a much slower reaction, the flavylium cation is attacked by water in position 2 leading to the hemiketal (**B**) (some years ago denominated pseudo-base). The rate of this reaction has a characteristic pH dependence, because it decreases by increasing pH. Brouillard and Dubois [2] explained this behaviour during the seventies of the last century: quinoidal base is a kinetic product motivated by the fact that this species does not hydrate in moderately acidic medium. In other words, formation of **B** (and the subsequent species **Cc** and **Ct**) takes place exclusively from the water attack to **AH^+^**. Consequently, higher pH values mean less flavylium cation available to react resulting in a decrease of the respective disappearance rate. On the other hand, the species **B** is converted in *cis*-chalcone (**Cc**) by a ring opening that occurs in sub-seconds and, finally, **Cc** gives *trans*-chalcone (**Ct**) by isomerization, a process that could occur in a few seconds or hours and even days. It should be noted that the *cis*-*trans* isomerism is referred to by the relative positions of the A and B rings (see Scheme 1). This terminology is more convenient than the *E*-*Z* isomerism (frequently used in anthocyanins) for comparing 3-substituted with 3-H compounds because in the first case the *cis* isomer corresponds *E* and in the last case to *Z*. In summary, in anthocyanins, three kinetic processes are well separated in time, the first step is proton transfer, the second step is a succession of two reactions, then hydration followed by tautomerization, being the former the rate limiting step. Noteworthy, at low pHs, the hydration can be faster, but this “change of regime” is only observed in reverse pH jumps, or from flash photolysis at sufficiently acidic solutions. Finally, the third step is the *cis*-*trans* isomerization that defines the kinetic approach to study the system as will be described below.

### 1.2. The Flavylium-Based Multistate

**Scheme 2:**
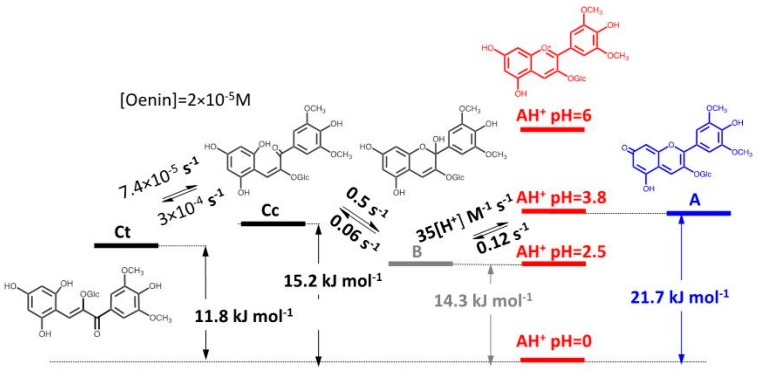
Energy-level diagram for malvidin-3-glucoside (Oenin), obtained according to the procedure shown in Appendix A [9]. The rate of the tautomerization reaction is faster than the values reported due to the acid and basic catalysis [3] (not included in the Scheme).

A very useful way to rationalize the chemistry of anthocyanins and related compounds is the construction of an energy level diagram as the one shown in Scheme 2 for malvidin-3-glucoside (oenin). The energy level diagram is easy to construct provided that the equilibrium constants of the four chemical reactions have been previously calculated, see Appendix A [9]. According to Scheme 2, **AH^+^** is the stable species at lower pH values. After a direct pH jump, for example to pH = 3.8, half of **AH^+^** and **A** are formed and remain in this fraction during the successive kinetic steps. However, **B** is at this pH value the most stable species and the system evolves to give **B** in fast equilibrium with the minor species **Cc**. Due to the fact that anthocyanins exhibit a high *cis*-*trans* isomerization barrier, at this point of the kinetics, a pseudo-equilibrium can be considered, a transient sate involving all species except **Ct**. The system reaches the equilibrium by formation of a small fraction of **Ct**, a process that takes a few hours.

One interesting characteristic of the flavylium based multistates is the great dependence of the relative energy levels of the different species according to the nature and position of the substituents in the flavylium core. For example, in 4′,7-dihydroxyflavylium, the species **Ct** has a lower energy and **B** and **Cc** are not detected at the equilibrium. They are transient species necessary to convert **AH^+^** into **Ct**, during the kinetic steps [9]. Conversely, in the case of 4-methyl-7-hydroxyflavylium, the equilibrium in water is established exclusively between **AH^+^** and **A**.

The reactions reported in Scheme 1 can be written in Equations (1)–(4):

*Proton transfer*

(1) $${\mathrm{AH}}^{+}+\;H_{2}O⇌{A\;+\;H}_{3}O^{+}\;K_{a}$$

*Hydration*

(2) $${\mathrm{AH}}^{+}{+\;2H}_{2}O⇌{B\;+\;H}_{3}O^{+}\;K_{h}$$

*Tautomerization*

(3) $$B\;⇌\mathrm{Cc}\;K_{t}$$

*Isomerization*

(4) $$\mathrm{Cc}\;⇌\mathrm{Ct}\;K_{t}$$

In spite of the complexity of the multi-equilibria, the flavylium multistate system can be simplified considering a single acid-base reaction involving the species **AH^+^** and a conjugate base **CB** defined as the sum of the other species **A**, **B**, **Cc** and **Ct**, Equation (5),

(5) $${\mathrm{AH}}^{+}{+\;H}_{2}O⇌{\mathrm{CB}\;+\;H}_{3}O^{+}\;K\prime_{a}$$

with [**CB**] = [**A**] + [**B**] + [**Cc**] + [**Ct**].

The equilibrium constant of Equation (5) relates with the equilibrium constants in Scheme 1 according to Equation (6).

(6) $$K\prime_{a}=K_{a}+K_{h}+K_{h}K_{t}+K_{h}K_{t}K_{i}$$

The pseudo-equilibrium is similarly given by Equations (7) and (8).

(7) AH++ H2O⇌CB^ + H3O+  K^a

with [**CB^^^**] = [**A**] + [**B**] + [**Cc**].

The apparent equilibrium constant of Equation (7) relates with the equilibrium constants in Scheme 1 according to Equation (8).

(8) K^a=Ka+Kh+KhKt

The kinetic processes following a direct pH jump (for the multistate systems exhibiting high *cis*-*trans* isomerization barrier as anthocyanins) are summarized in Equations (9)–(11) [10].

Equation (9) regards the first kinetic step that converts **AH^+^** into **A**. This equation and the subsequent have been deduced taking into account the reversibility. In other words, the observed rate constant in such a case is the sum of the direct and reverse rate constants.

(9) $$k_{1}=k_{a}+k_{-a}\left[H^{+}\right]$$

Considering the second process, the rate determining step is the hydration and thus the direct rate constant should be multiplied by the mole fraction of **AH^+^**, *X*_AH+_, (from the acid base equilibrium with **A**) and the reverse rate constant by the mole fraction of **B** available, *X*_B_, (from the tautomerization equilibrium with **Cc**)

(10) $$k_{2}=X_{AH^{+}}k_{h}+X_{B}k_{-h}\left[H^{+}\right]=\frac{\left[H^{+}\right]}{\left[H^{+}\right]+K_{a}}k_{h}+\frac{1}{1+K_{t}}k_{-h}\left[H^{+}\right]$$

When there is a high *cis*-*trans* isomerization barrier, the system reaches a pseudo equilibrium where all the species **AH^+^**, **A**, **B** and **Cc** can be considered in (pseudo)equilibrium, because their interconversion is much faster than the isomerization, that in anthocyanins takes a few hours. In the kinetics of this last step, the direct rate constant *k*_i_ should be multiplied by the mole fraction of **Cc,** *X*_Cc_, involved in the pseudo-equilibrium available to isomerize. See Appendix B for the deduction of the mole fractions at the equilibrium and pseudo-equilibrium [9].

(11) $$k_{2}=X_{Cc}k_{i}+k_{-i}=\frac{K_{h}K_{t}}{\left[H^{+}\right]+K_{a}+K_{h}+K_{h}K_{t}}k_{i}+k_{-i}$$

**Scheme 3:**
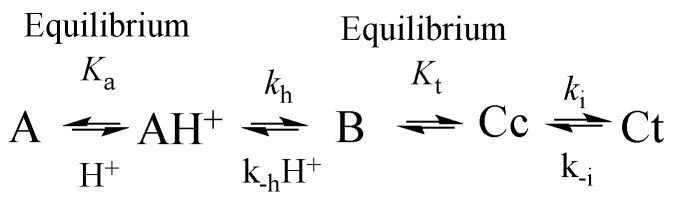
Flavylium network for compounds with small isomerization barrier: **A**/**AH^+^** and **B**/**Cc** are in fast equilibrium.

In many other flavylium networks, the *cis*-*trans* isomerization barrier is very small, the pseudo-equilibrium is not formed and steps 2 and 3 described above are transformed into one step (Scheme 3). Assuming that the equilibrium between **AH^+^** and **A** as well **B** and **Cc** is reached during the kinetic process, the following Equation (12) can be deduced, again from simple reasoning without use of more complex mathematical deductions [10].

(12) $$k_{4}=\frac{\frac{\left[H^{+}\right]}{\left[H^{+}\right]+K_{a}}K_{h}K_{t}k_{i}+k_{-i}\left[H^{+}\right]}{\left[H^{+}\right]+\frac{K_{t}k_{i}}{k_{-h}}}$$

## 2. Results and Discussion

### 2.1. Low cis-trans Isomerization Barrier

**Scheme 4:**
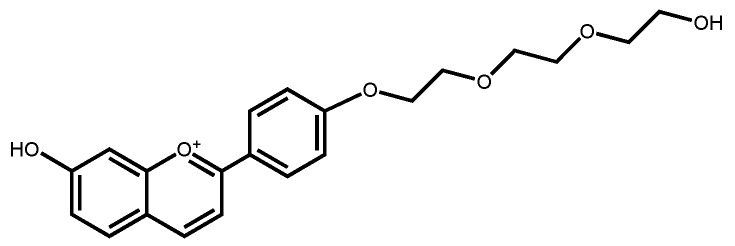
Compound **1**.

**Figure 1:**
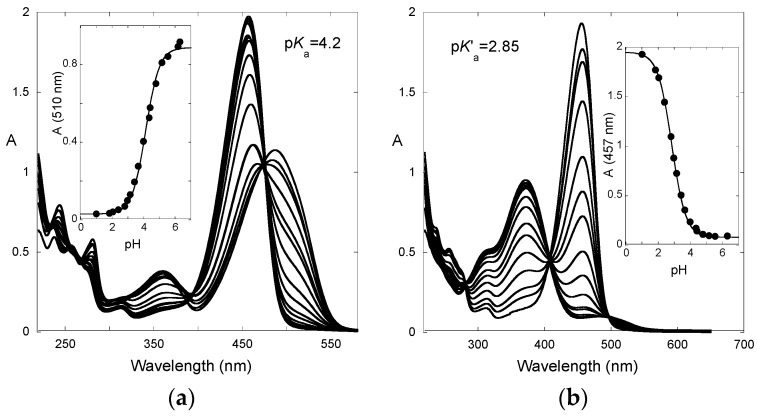
(**a**) Absorption spectra of compound **1** taken immediately (circa 1 min) after a direct pH jump; (**b**) the same at the equilibrium.

The equilibrium between **AH^+^** and **A**, Equation (1), is illustrated for the compound shown in Scheme 4 and Figure 1a. The absorption spectra were taken immediately after the pH jump before significant disappearance of **AH^+^**/**A**.

The spectral variations are typical of anthocyanins and related compounds bearing hydroxyl substituents, showing isosbestic points and the absorption of the quinoidal base red shifted in comparison with flavylium cation. The measured p*K*_a_ = 4.2 is the same of the analogous compound 7-hydroxy-4′-methoxyflavylium [11]. In Figure 1b, the absorption spectra of the same solutions at the equilibrium are presented, Equation (5). In this case, the flavylium cation is involved in a single acid base equilibrium with **CB** (basically **Ct** and a minor contribution from **A**, **Cc** and **B** being negligible). The characteristic absorption spectrum of **Ct** appears blue shifted and the isosbestic points confirm that the system at the equilibrium is equivalent to a single acid base reaction with acid-base constant equal to p*K*′_a_ = 2.85, which compares with 3.0 for 7-hydroxy-4′-methoxyflavylium [11]. 

**Figure 2:**
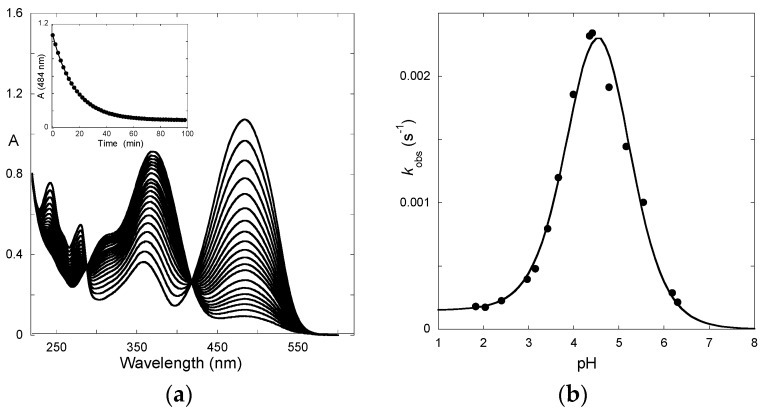
(**a**) Spectral variations after a direct pH jump 1–5.5; *k*_obs_ = 1 × 10^−3^ s^−1^; (**b**) Representation of the pH dependent rate constants of the kinetic process to reach the equilibrium after a direct pH jump. Fitting was achieved by means of Equation (12) for the parameters reported in Table 1.

Compound **1**, similarly to 7-hydroxy-4′-methoxyflavylium, does not have a high *cis*-*trans* isomerization barrier and by consequence from the situation reported in Figure 1a to the one in Figure 1b, only one kinetic process is observed, as shown in Figure 2a. Representation of the rate constants as in Figure 2a for different pH values can be fitted with Equation (12), see Figure 2b and Table 1.

According to Table 1, and Figure 1 and Figure 2, the following relationship between the equilibrium constants is known *K*_a_ + *K*_h_ + *K*_h_*K*_t_ + *K*_h_*K*_t_*K*_i_ = 10^−2.85^, *K*_a_ = 10^−4.2^.

The relations of the constants are not enough to calculate all the constants and further experiments should be done.

### 2.2. A Little Help from Photochemistry

**Scheme 5:**
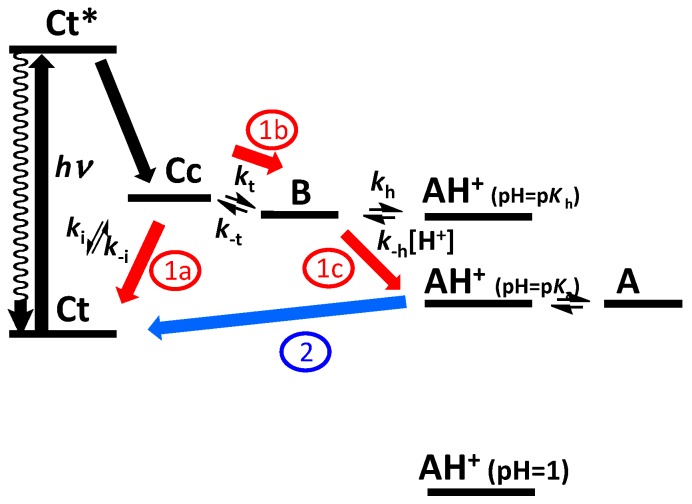
Representation of the photochemical process in flavylium multistates.

When the *trans*-chalcone is the major species of the conjugated base, **CB**, there is the possibility of using light to create a perturbation in the system and follow the associated kinetic processes. The procedure is shown in Scheme 5. Excitation of the *trans*-chalcone gives rise to the *cis* isomer. This last species could give backward the *trans*-chalcone or forward flavylium cation/quinoidal base via tautomerization and dehydration (step 1). The light irradiation is thus accompanied by the appearance of the coloured species flavylium cation and/or quinoidal base depending on pH and p*K*_a_ of the system. In a second process (step 2), the system equilibrates and this kinetic step is equivalent to a direct pH jump to the pH of the irradiation.

In previous work, the quantum yields as a function of pH were used to obtain the relations between rate and equilibrium constants in order to find an independent set of equations to resolve the system [12]. In this work, we report for the first time an alternative method, which is easier and more precise than the quantum yields.

### 2.3. Flash Photolysis in Flavylium Systems

**Figure 3:**
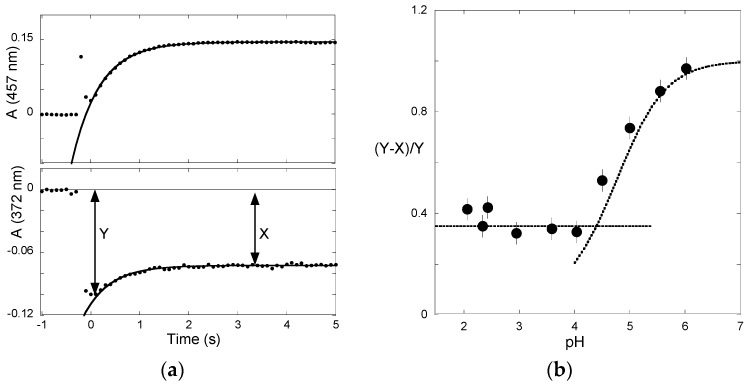
(**a**) Flash photolysis traces after a light flash at pH = 4.04.; (**b**) Ratio (Y − X)/Y corresponding to the efficiency of the back reaction as a function of pH. A global fitting was carried out using the data from Figure 1, Figure 2 and Figure 3.

Some years ago, we reported a simple and inexpensive flash photolysis apparatus built with a flash camera and a spectrophotometer in time drive [13]. Currently, a system of optical fibbers is used. The time scale of the experiments is a few seconds, the most convenient timeframe to follow the kinetics of step 1, in Scheme 5. Figure 3a shows the absorption traces monitored at 372 nm and at 475 nm after the flash pulse. These wavelengths correspond to the absorption maximum of **Ct** and **AH^+^/A**, respectively. The observed rate constant (equal at both wavelengths) is the sum of the forward and backward rates, accounting to two parallel reactions that are responsible for the disappearance of **Cc** to give **Ct** (backward reaction) and **AH^+^/A** (forward reaction). 

The absorbance variations of Figure 3a observed immediately after the flash pulse clearly show a bleaching of the **Ct** absorption at 372 nm, because the molar absorption coefficient of **Ct** is higher than **Cc** at this wavelength. Immediately after the flash, no **AH^+^/A** is formed. The amplitude Y is proportional to the amount of **Ct** that disappeared to give **Cc**. However, at this pH, a fraction of **Ct** (Y − X) is recovered. This result indicates a low *cis*-*trans* isomerization barrier and that in less than 2 seconds part of **Cc** goes back to **Ct**. Simultaneously, the fraction X of **Ct** lost is the one that is responsible for the formation of **AH^+^/A** observed at 457 nm. Representation of the efficiency of the backward process η*_Ct_* = (Y − X)/Y is reported in Figure 3b. According to Figure 3b, there is a change of the kinetic regime around pH 4.5. This behaviour was already observed for the quantum yield pH dependence. At lower pH values, the hydration that is dependent on the proton concentration becomes faster than tautomerization (change of regime) [14]. The kinetics after the change of regime (lower pH values) can be accounted for by Equation (13). The equilibration between **B** and **Cc** does not occur because once **B** is formed it immediately disappears to give **AH^+^/A** (i.e., the tautomerization is the rate limiting step).

(13) $$k_{flash\left(low\;pH\right)}=k_{i}+k_{-t}+k_{-t}^{H}\left[H^{+}\right]+k_{-t}^{OH}\left[{\mathrm{OH}}^{-}\right]$$

where the terms $k_{-t}^{H}$ and $k_{-t}^{OH}$ refer to the acid and basic catalysis of the tautomerization, respectively.

For the present compound, the basic catalysis is not significant at this pH range and *k_i_* is much higher than *k_−i_* allowing the simplification of this equation ($k_{i}+k_{-t}+k_{-t}^{H}\left[H^{+}\right]$).

The efficiency of **Ct** recovery is thus given by Equation (14)

(14) $$\eta_{Ct}=\frac{k_{i}}{k_{i}+k_{-t}+k_{-t}^{H}\left[H^{+}\right]}$$

At higher pH values, the process is controlled by the hydration reaction, and so there is time to establish the equilibrium between **B** and **Cc**. By consequence, the disappearance of **B** and **Cc** is multiplied by the respective mole fractions *X*_B_, and *X*_Cc_, and the same holds for the flavylium cation which must be multiplied by *X*_AH+_, Equation (15), because **AH^+^** and **A** are in fast equilibrium.

(15) $$\begin{array}{cc} k_{flash\left(high\;pH\right)}= & X_{B}k_{-h}\left[H^{+}\right]+X_{AH^{+}}k_{h}+X_{Cc}k_{i}\\ & =\frac{\left[H^{+}\right]}{1+K_{t}}k_{-h}+\frac{\left[H^{+}\right]}{\left[H^{+}\right]+K_{a}}k_{h}+\frac{K_{t}}{1+K_{t}}k_{i} \end{array}$$

The efficiency of the **Ct** recovery is thus given by Equation (16).

(16) $$\eta_{Ct}=\frac{\frac{K_{t}}{1+K_{t}}k_{i}}{\frac{\left[H^{+}\right]}{1+K_{t}}k_{-h}+\frac{\left[H^{+}\right]}{\left[H^{+}\right]+K_{a}}k_{h}+\frac{K_{t}}{1+K_{t}}k_{i}}$$

**Scheme 6:**
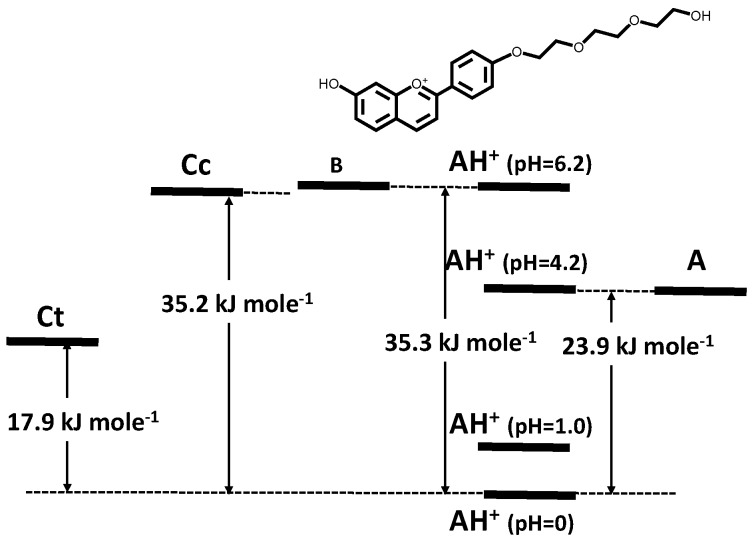
Energy level diagram of compound **1**.

A global fitting of Equations (14) and (16), Figure 3b together with the data from Table 1 allow calculating all the equilibrium (Table 2) and rate (Table 3) constants of the multistate and drawing the respective energy level diagram, Scheme 6.

### 2.4. High cis-trans Isomerization Barrier

The kinetics of flavylium multistates exhibiting a high *cis*-*trans* isomerization barrier, as observed in anthocyanins, requires a different kinetic treatment and the compounds do not need to be photochromic to access all kinetic and thermodynamic constants. Because the isomerization step is very slow, it is possible to obtain a transient state (pseudo-equilibrium) which behaves like the equilibrium but the apparent acid-base constant *K*^^^_a_ is equal to *K*_a_ + *K*_h_ + *K*_h_*K*_t_. The constants *K*_a_, *K*^^^_a_ = *K*_a_ + *K*_h_ (1 + *K*_t_) and *K*′_a_ = *K*_a_ + *K*_h_ (1 + *K*_t_ + *K*_t_*K*_i_) lead to a system of three equations of four unknowns. Calculation of *K*_t_ through reverse pH jumps, see below, allows solving the system and obtaining all the equilibrium constants, allowing the construction of the energy level diagram.

**Scheme 7:**
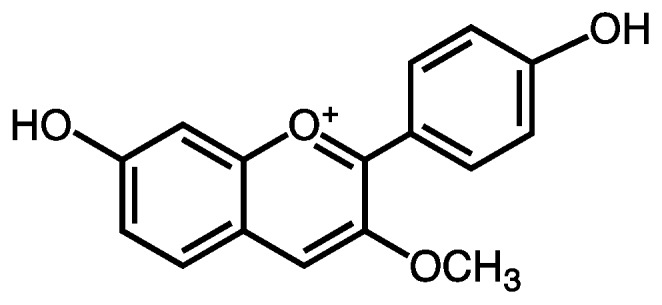
Compound **2**.

**Figure 4:**
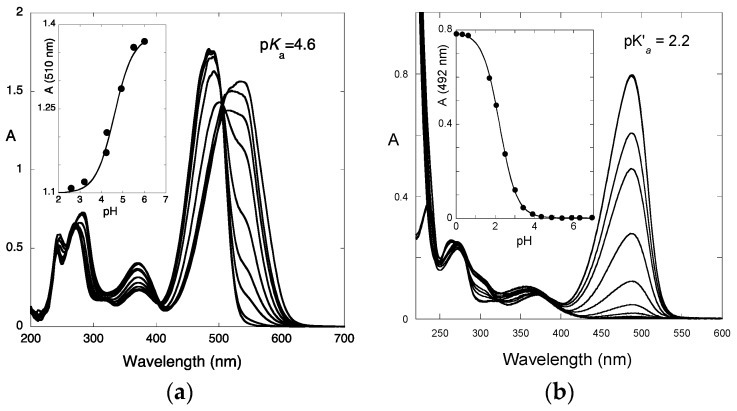
(**a**) Spectral variations immediately after a direct pH jump for compound **2**; (**b**) the same at the equilibrium. With permission from RSC from ref. [15].

The model compound **2** (Scheme 7) is used to illustrate the procedure for the compounds bearing high *cis*-*trans* isomerization barrier. A communication regarding its dual photochromism was recently reported [15]. In this work, the experimental data necessary to calculate the rate and equilibrium constants as well as the energy level diagram of this multistate were taken from the appendix of this work [15]. Figure 4 shows the pH-dependent spectral variation immediately after the pH jumps (a) and at the equilibrium (b).

**Figure 5:**
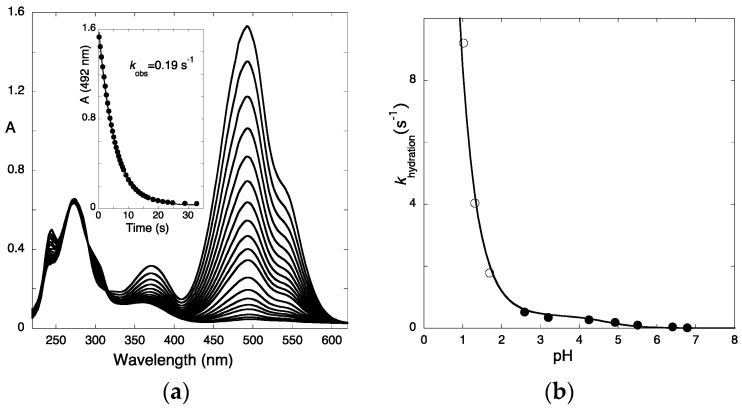
(**a**) Spectral variations observed upon a direct pH jump from 1 to 4.9; (**b**) pH dependence of the kinetic process controlled by the hydration reaction followed by stopped flow: (•) direct pH jumps; Fitting was achieved with Equation (10) for p*K*_a_ = 4.6; *k*_h_ = 0.3 s^−1^; *k*_-h_ = 75 M^−1^·s^−1^. (°) reverse pH jumps, fitting was achieved with Equation (17) which is coincident with Equation (10) within experimental error due to the low value of *K*_t_ = 0.05, see below. With permission of RSC from ref. [15].

The flavylium multistates possessing a high *cis*-*trans* isomerization barrier have a great advantage, because the three kinetic processes occur in very different time scales and can be treated separately, see Equations (10) and (11) of the introduction. The calculation of the rate constants can be achieved by following the kinetics of the direct and reverse pH jumps. In Figure 5a, the kinetics of the second process taking place upon a direct pH jump is shown. The quinoidal base in equilibrium with flavylium cation formed during the mixing time of the base disappears in some seconds to give the pseudo-equilibrium state. At this pH (4.9), the hydration is the rate determining step, meaning that as soon as **B** is formed, it equilibrates with **Cc**. In Figure 5b, the rate constants of this step are plotted as a function of pH and were fitted by Equation (10).

The fitting of Equation (10) allows obtaining the value of *k*_h_ = 0.3 s^−1^ (*K*_a_ was already calculated from Figure 4a) and the ratio *k*_−h_/(1 + *K*_t_) = 71.4 M^−1^·s^−1^. At this point, it is clear that the equilibrium constant *K*_t_ is a key constant that is needed to calculate all the equilibrium and rate constants, see below.

### 2.5. Reverse pH Jumps

**Figure 6:**
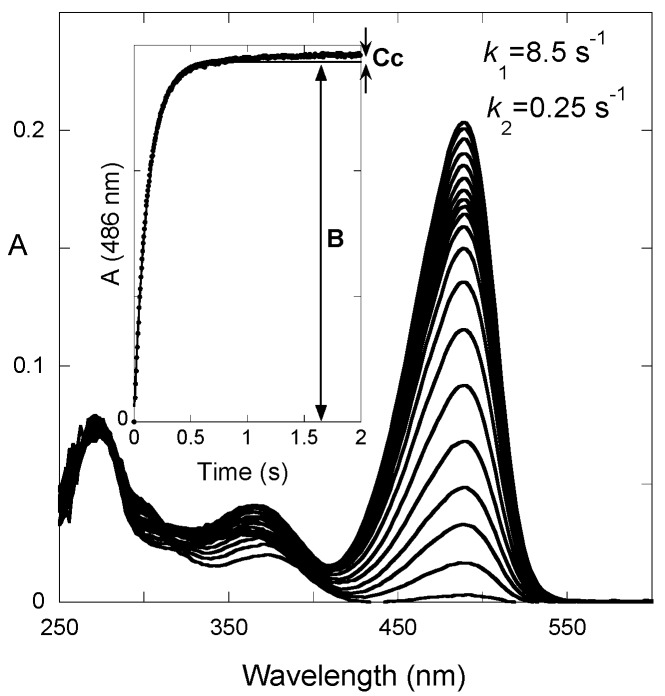
Spectral variations upon a reverse pH jump of the compound 4′,7-dihydroxy-3-methoxyflavylium 7.5 × 10^−6^ M, from equilibrated solutions at pH = 6 to 1, *K*_t_ = 0.05. With permission from RSC, ref. [15].

The reverse pH jumps to study the flavylium systems were introduced by McClelland and co-workers [3,16,17]. It consists of two steps: (i) the first one to reach the equilibrium (or pseudo equilibrium) after a direct pH jump, (from the flavylium cation); (ii) the second one back to the flavylium cation by addition of acid and the respective spectral variations collected using a stopped flow equipment (Figure 6).

At higher proton concentration, the hydration becomes faster than tautomerization and by consequence the flavylium cation absorption increases according to two kinetic processes. The first one corresponds to the conversion of **B** into **AH^+^** and the second and slower to the conversion of **Cc** into more **AH^+^** via **B**. Equations (17) and (18) account for these two steps. Moreover, the ratio of the amplitudes of the slowest and faster exponentials is equal to *K*_t_ = [**Cc**]/[**B**].

(17) $$k_{reverse1}=\frac{\left[H^{+}\right]}{\left[H^{+}\right]+K_{a}}k_{h}+k_{-h}\left[H^{+}\right]$$

(18) $$k_{reverse2}=k_{-t}$$

The accuracy of this method is very dependent on the fraction of **B** and **Cc**. In anthocyanins and in the present model compound, the fraction of **Cc** is very small (*K*_t_ = 0.05) and several experiments should be carried out to give some statistical meaning to the constant. In principle, the pH dependence of the hydration rate constants obtained from direct pH jumps, Equation (10), is different from the one obtained from reverse pH jumps, Equation (17). In the present compound, they are practically coincident due to the very small value of *K*_t_, see Figure 6.

At this point, all the equilibrium constants can be calculated. Regarding the rate constants *k*_−h_ is obtained from Equation (10) and/or Equation (17) and *k*_h_ from the equilibrium constant *K*_h_. The same for *k*_−t_ Equation (18) and *k*_t_ from *K*_t_.

**Figure 7:**
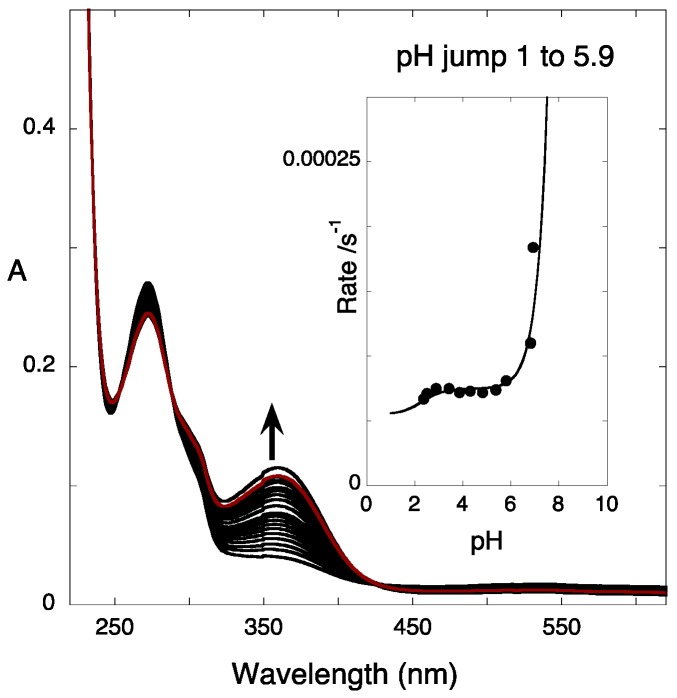
Spectral variations regarding the slowest step to attain the equilibrium, which is controlled by the *cis*-*trans* isomerization. The increase in the rate for pH > 6 take place from the ionized species and was not used in this calculation, which restrains to the moderately acidic pH range. With permission from RSC reference [15].

Going back to the direct pH jumps, the last step to attain the equilibrium is the formation of **Ct** through a much slower process, Figure 7.

In the pH region up to pH = 6, the fitting in the inset is achieved with Equation (11) allowing calculation of the remaining rate constants *k*_i_ and *k*_-i_. A final control should be made, to assure the coherence of the constants, see Table 4 and Table 5.

**Scheme 8:**
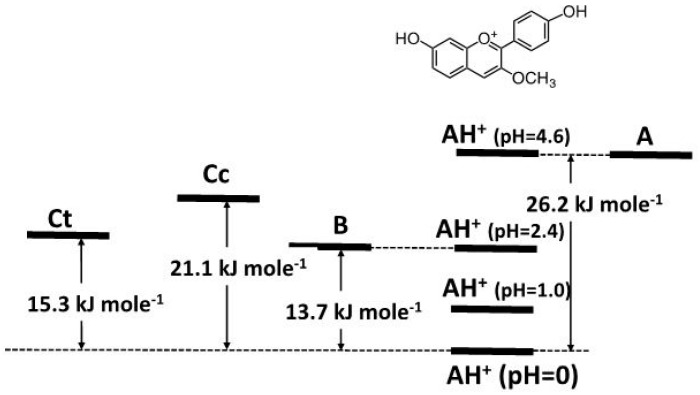
Energy level diagram of compound **2**.

**Scheme 9:**
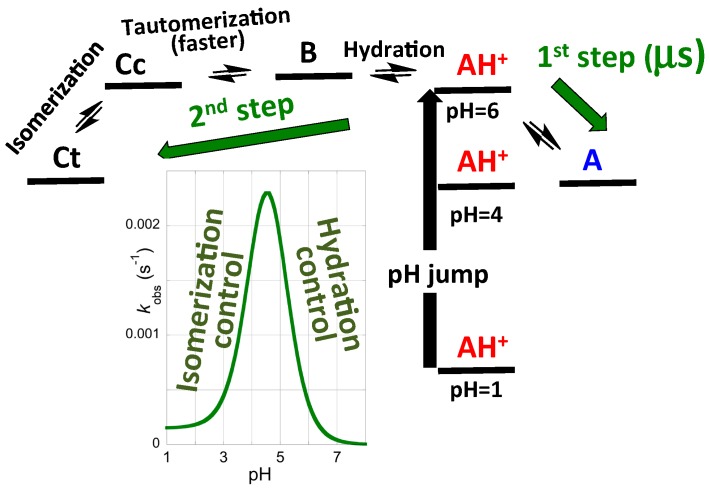
Energy level diagram of a system with low *cis*-*trans* isomerization barrier. During the mixing time, an equilibrium between **AH^+^** and **A** is established and the kinetics towards the equilibrium is constituted by a single step.

**Scheme 10:**
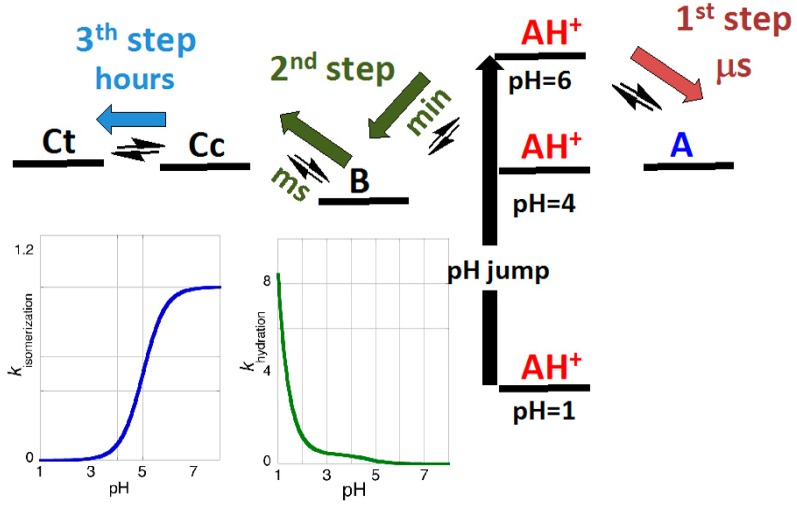
Energy level diagram of a system possessing high *cis*-*trans* isomerization barrier. During the mixing time, an equilibrium between **AH^+^** and **A** is established. The second step (green) is controlled by the hydration reaction, and the third and slower (blue) by the isomerization.

Comparing the energy level diagram of compounds **1** and **2**, respectively Scheme 6 and Scheme 8, the most dramatic effect is the much higher energy level of species **B** and **Cc**, in the former. Substitution in position 3 by a methoxy, a model compound of anthocyanins, stabilizes the species **B** in agreement with the observed behaviour of anthocyanins. In Scheme 8, the energy level diagram and the respective kinetic patterns are shown for the two types of kinetics. Considering a direct pH jump, in both cases the proton transfer is the faster reaction, leading to an acid base equilibrium between flavylium cation and quinoidal base. These two species behave in the subsequent kinetic steps as a single one. In other words, disappear simultaneously and maintain the fraction **AH^+^**/**A** equal to [H^+^]/*K*_a_. When there is a low isomerization barrier, the equilibrium is reached through a single step, which presents a bell shape variation with pH, Scheme 9. Conversely, in the presence of a high barrier, two kinetic steps could be identified, the hydration (followed by fast tautomerization) and the slower isomerization, Scheme 10.

### 2.6. Applications

#### 2.6.1. Host–Guest Interaction with a Flavylium Based Multistate

**Scheme 11:**
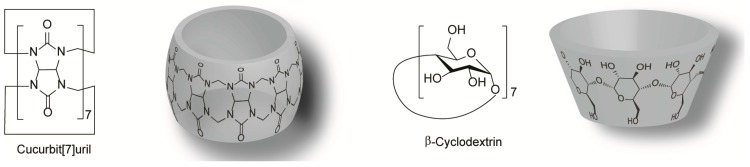
Structures of cucurbit[7]uril and β-cyclodextrin.

The co-pigmentation is a very important issue in the expression of the colour in plants [18]. In spite of co-pigmentation being a complex process, it is possible to conceive a simple model to account for the basic physical chemical processes that are associated with it. In this context, host–guest complexes involving cucurbit[7]uril (CB7) and β-cyclodextrins (β-CD), Scheme 11, are interesting examples to give some clues regarding co-pigmentation interactions in vivo.

**Scheme 12:**
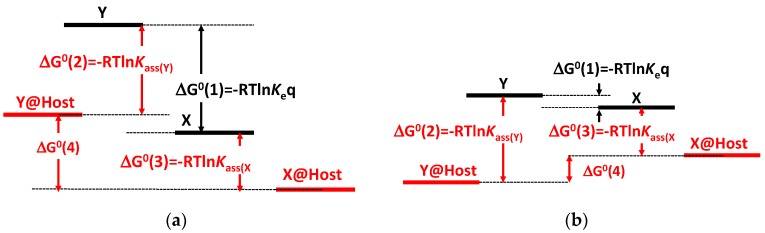
(**a**) Energy level diagram regarding the equilibrium Y ⇌ X, with X slightly stable than Y. Inversion of stability occurs upon interaction with the host; (**b**) The association constants are the same as A, but X is much more stable than Y. Inversion of stability does not take place.

Considering an energy level diagram of a flavylium-based multistate, the mole fraction distribution of the adducts would change as a function of the magnitude of the association constant and this is reflected in the relative changes in the diagram. Let us consider two species of the multistate with equilibrium constant *K*_eq_, assuming a 1:1 interaction, and an association constant *K*_ass_ (Scheme 12).

In the example of Scheme 12a, the species X is slightly more stable than the species Y. If the association with Y is much more efficient than with X, there is inversion of the stability and the complex with Y becomes more stable than the one with X. However, if X is much more stable than Y, Scheme 12b, unless the association with Y is very much stable than with X the inversion does not take place. 

In conclusion, for the multistate species, that in the absence of the host have a high energy level (their mole fraction distribution at the equilibrium is very small), difficultly would become significant upon host–guest interaction.

#### 2.6.2. Cucurbit[7]uril

**Scheme 13:**
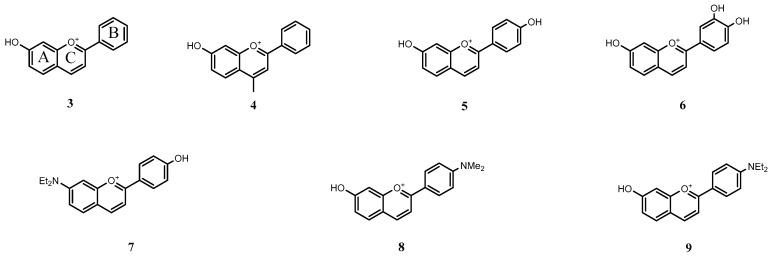
Flavylium derivatives employed as guest molecules to form inclusion complexes with cucurbit[7]uril.

Cucurbit[n]urils are recognized for their ability to complex positively charged molecules with high affinity and thus it was not surprising to find that flavylium cations form inclusion complexes with CB7. The stability of the complexes with several flavylium derivatives (Scheme 13 and Table 6) was investigated by absorption and/or emission spectroscopy while their structural properties were elucidated by ^1^H-NMR [19]. All compounds were found to form 1:1 complexes with this host and the association constants vary between *K* = 2.7 × 10^5^ M^−1^ for compound 6 and *K* = 8.0 × 10^6^ M^−1^ for 8. The results suggest that hydrophobicity, dimension and the nature of the substituent affect the stability for CB7.

The nature and position of the substituents in the flavylium core have only a moderate influence on the stability of the complexes. On the other hand, the inclusion mode seems to be strongly dictated by the substituents. For compounds **3** and **4**, the B ring is specifically included within the host cavity while, in the cases of **5** and **6**, the ^1^H-NMR experiments suggest an unspecific dynamic behaviour with the CB7 shuttling quickly between the AC and B rings. This behaviour was more pronounced and, thus, more evident in the case of **6** because, for **5**, despite evidence of dynamic shuttling, the B ring seems to be favoured and more populated.

In the case of the amino-substituted compounds (**7**, **8** and **9**), these groups were shown to dictate the structure of inclusion complexes. The partially positive character of the nitrogen atom of these groups, due to electronic delocalization, allows attractive ion–dipole interaction with the carbonyl oxygen atoms of the host, resulting in favoured inclusion of the B ring in the case of **8**. On the other hand, for the diethylamino substituent it was found that independently of his position (A or B ring), evidence for deep inclusion of this group in the CB7 cavity was found in both cases probably due to its optimal packing coefficient of 55% [20]. Nevertheless, it is worth mentioning that, for **9**, the experimental results suggest the existence of dynamic behaviour with the host shuttling between the diethylamino and the A and C ring motifs. 

**Figure 8:**
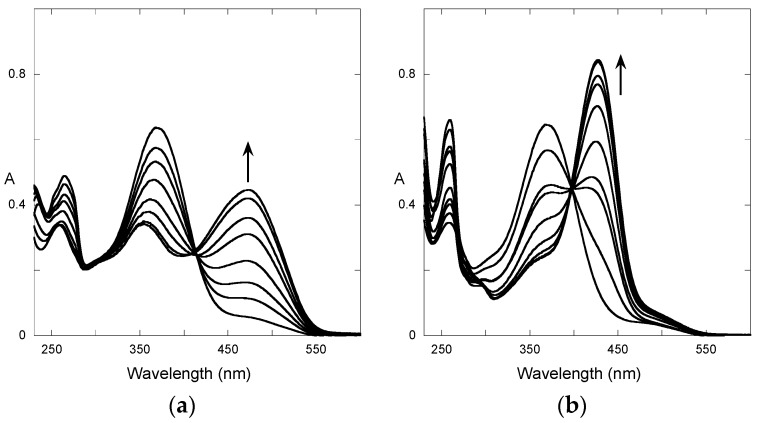
Equilibrated spectra of compound **3** (7-hydroxyflavylium) in the presence of different concentrations of CB7 (up to 2.5 mM) at pH 6 (**a**); and pH 4 (**b**). With permission from ACS reference [22].

The high affinity of CB7 for the flavylium cation can be leveraged to increase the stability of this coloured species in less acidic conditions. In the case of the trihydroxy derivative **6**, a shift in the p*K*′_a_ from 3.1 to 4.3 in the presence of 0.3 mM of CB7 was observed [21]. This results can be easily rationalized by taking into account the energy diagram of Scheme 12 and arise from the preferential stabilization of the flavylium cation in comparison with neutral species. Our attempts for colour stabilization were more impressive in the case of 7-hydroxyflavylium **3** [22]. For this compound, a p*K*′_a_ shift from 2.3 to 4.8 was observed for 1 mM of host. This can be ascribed, mainly, to higher affinity of CB7 for **3** comparatively with **6** (*K* is almost one order of magnitude higher) and also to higher concentrations of host employed in the last example. More interesting (and unpredicted) was the fact that amongst the neutral species, CB7 also showed selectivity for the coloured quinoidal base. Thus, increasing the concentration of host to a solution of **3** equilibrated at pH 6 (mainly composed by uncoloured *trans*-chalcone) drives the equilibrium towards the formation of quinoidal base (Figure 8a). Likely, when CB7 is added to a solution of **3** at pH 4, the equilibrium is driven to the regeneration of the flavylium cation (Figure 8b), demonstrating the ability of this host to selectively bind and stabilize the coloured species of the multistate.

The potential of CB7 to stabilize the flavylium species was also investigated by Bohne and Quina, who have showed that 3′,4′,7-trimethoxyflavylium (p*K*′_a_ = 3.0) is completely stabilized at pH = 5.5 in the presence of low concentrations of CB7 (0.12 mM) [23]. The authors found evidences for the formation of 2:1 host–guest complexes that can be responsible for this remarkable stabilization (it requires a p*K*′_a_ > 7.0 to keep the mole fraction of flavylium higher than 97% at this pH).

#### 2.6.3. 6,8 Rearrangement

The 6,8 rearrangement was previously described by Jurd and Andersen, in acidic pH values [24,25]. More recently, it was extended to the other species of the multistate [26].

**Scheme 14:**
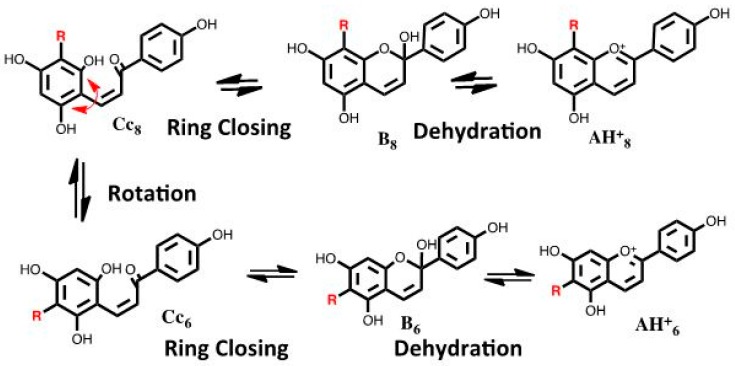
Key step of the 6,8 rearrangement in flavylium multistates.

The 6,8 rearrangement consists of the formation of a flavylium cation having a substituent in position 8 that migrates to position 6 and vice-versa, see Scheme 14. In order to observe this isomerization, the flavylium cation should possess a hydroxyl in position 5 and the substituents in position 6 and 8 should be different. This is the reason why 6,8 rearrangements are not observed in anthocyanins monoglucoside and deoxyanthocyanins, (like luteolinidin), because both positions 6 and 8 are substituted by H.

**Figure 9:**
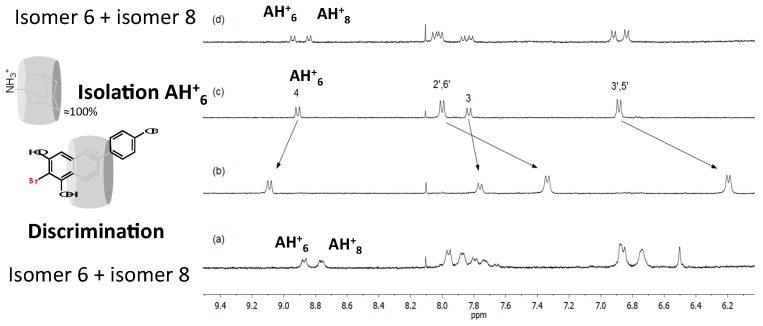
^1^H-NMR spectra of the compound 6-bromo apigeninidin and its isomer **8** (**a**) at the equilibrium pH = 1.0; (**b**) at the equilibrium after addition of cucurbit[7]uril; (**c**) immediately after addition of 1-aminoadamantane (AD) (**d**) Equilibration of (**c**) which is coincident with (**a**).

The 6,8 rearrangement in the case of the compound **6** (or **8**)-bromo apigeninidin at the equilibrium, pH = 1.0, leads to circa 50% of each isomer [26]. It was possible to separate both isomers by HPLC and extend the study to the other species of the multistate. An alternative method to isolate (transiently) isomer **6** is through selective binding with CB7 [27]. As demonstrated by ^1^H-NMR experiments (Figure 9), in the presence of CB7 at pH = 1.0 the 6,8 rearrangement equilibrium is completely displaced toward the formation of isomer **6** owing to its selective binding and making it possible to isolate this species from the initial mixture with a rate constant *k*_obs_ = 1.3 × 10^−5^ s^−1^ Figure 9b. Addition of 1-aminoadamantane which presents a much higher association constant with CB7 (*K*_ass_ = 2 × 10^14^ M^−1^) [28] leads to the releasing of pure AH^+^_6_. This one slowly reverts back to 50% mixture of the equilibrium (d) coincident with (a).

#### 2.6.4. β-Cyclodextrin Interaction

While CB7 is efficient to selectively recognize the flavylium cation, the β-cyclodextrin binds preferentially the *trans*-chalcone. 

**Scheme 15:**
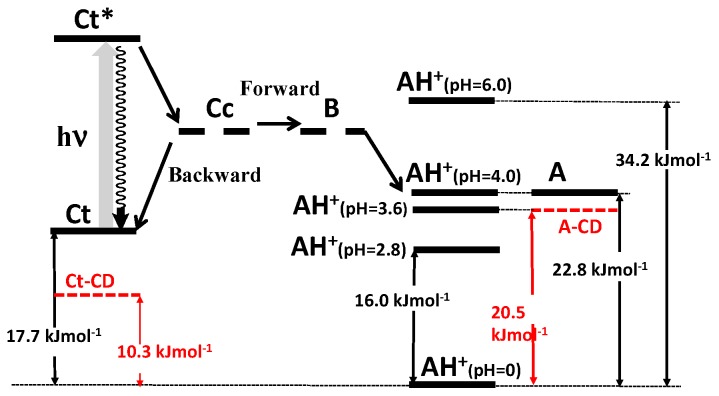
Energy level diagram of 4′,7-dihydroxyflavylium in the absence (black color) and presence of β-cyclodextrin 9 mM (red color). With permission of ACS from reference [29].

In Scheme 15, the interaction of β-cyclodextrin with the multistate of 4′,7-dihydroxyflavylium is shown [29]. The interaction of β-cyclodextrin with flavylium cation is negligible while the association constants with the quinoidal base and *trans*-chalcone are respectively 100 M^−1^ and 2.1 × 10^3^ M^−1^. In the absence of the host, the flavylium cation is in an acid-base equilibrium with *trans*-chalcone (87%) and quinoidal base (13%), hemiketal and *cis*-chalcone could not be detected at the equilibrium. In the presence of β-cyclodextrin 9 mMn these fractions change to 99% and 1%, respectively. The energy level diagram is once more very useful to visualize the system, Scheme 15.

**Scheme 16:**
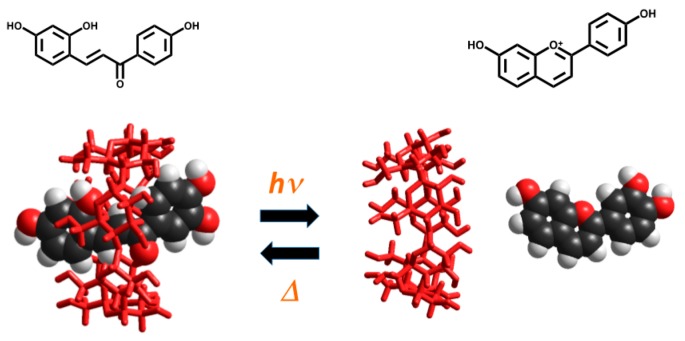
Emptying the β-cyclodextrin cavity from 4′,7-dihydroxyflavylium by the action of light.

Irradiation of the *trans*-chalcone inside the host leads to the flavylium cation that does not interact and is removed from the cavity (Scheme 16). The system is reversible and defines a photochromic system showing great potential to be applied in the fields of photo-responsive host–guest complexes and self-assembled soft materials.

**Figure 10:**
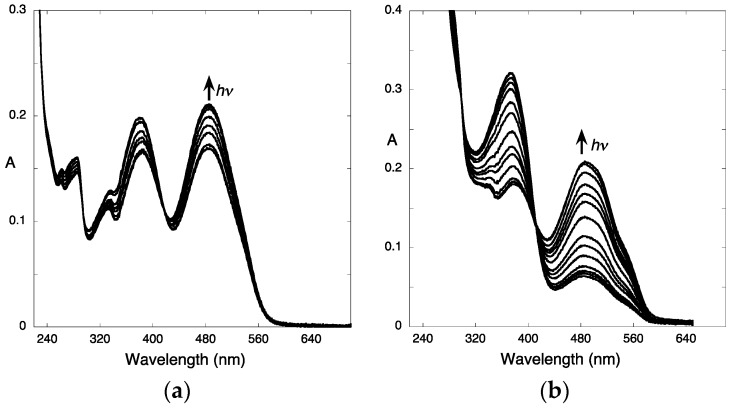
(**a**) Spectral variations upon irradiation at 366 nm of dracoflavylium (2 × 10^−5^ M) at pH = 4.1; (**b**) the same at pH = 3.1 in the presence of the HCD (1.6 × 10^−2^ M) illustrating the higher photochromic contrast observed in the presence of the host molecule. With permission from RSC, reference [30].

The formation inclusion complexes between (2-hydroxypropyl)-β-cyclodextrin and dracoflavylium (4′,7-dihydroxy-5-methoxyflavylium), a natural pigment extracted from a resin (Dragon’s blood) appearing in the injury parts of the tree Dracaena Draco, was investigated in a subsequent work [30]. Similarly to 4′,7-dihydroxyflavylium, the multistate of dracoflavylium at low acidic pH values is composed by quinoidal base and *trans*-chalcone species. However, in this case, the former species predominate (63%). The high fraction of colored quinoidal base at the equilibrium reduces the contrast of the photochromic system and, consequently, his potential applications. This problem was overcome by taking advantage of the preferential affinity of β-cyclodextrin to complex the *trans*-chalcone and as a result its fraction increases from 37% to 87% at the expense of the quinoidal base. This allowed an improvement in the performance of this photochromic system based on water-soluble natural products (Figure 10).

**Scheme 17:**
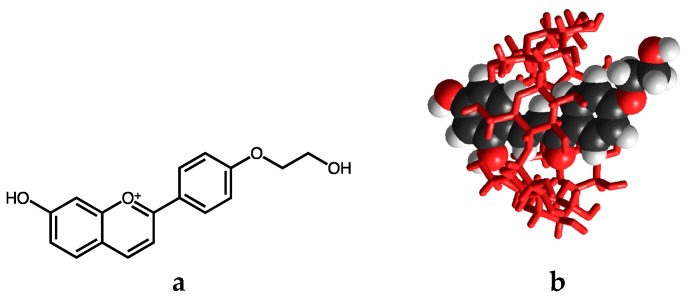
(**a**) 4′-(2-hydroxyethoxy)-7-hydroxyflavylium and (**b**) possible structure of the respective *trans*-chalcone in the presence of β-cyclodextrin.

**Figure 11:**
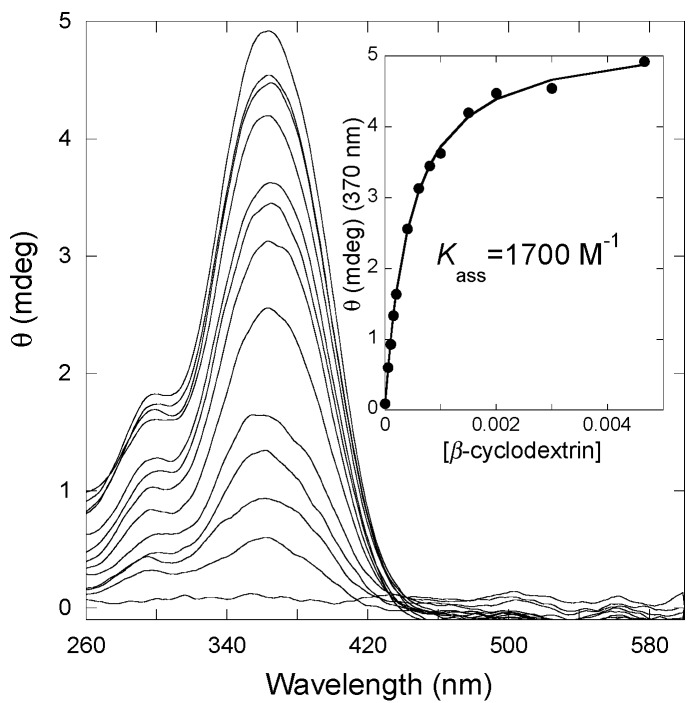
Circular dichroism spectra of the compound 4′-(2-hydroxyethoxy)-7-Hydroxyflavylium in the presence of increasing concentrations of β-cyclodextrin at pH = 4.8. With permission from Elsevier, reference [31].

Direct evidence for the inclusion of a *trans*-chalcone (of the multistate system based on 4′-(2-hydroxyethoxy)-7-hydroxyflavylium) in the β-cyclodextrin cavity was obtained from induced circular dichroism spectra, Figure 11 and Scheme 17 [31].

In Figure 11, the CD spectra as a function of β-cyclodextrin is shown. This type of signal is due to the induced circular dichroism (ICD) that occurs when a chiral transparent compound causes a structural perturbation or a coupling of the transition moments with achiral UV-Vis absorbing molecules [32]. The ICD is therefore a very powerful technique to investigate inclusion phenomena in multistate systems because only complexed species show ICD signals while the uncomplexed ones are silent. Owing to this advantage, it can be used to demonstrate (or discard) selective complexation and to measure association constants without perturbation from the other components of the system [33].

## 3. Experimental Section

### 3.1. General

All reactants and solvents obtained from commercial suppliers were used without further purification. The solutions were prepared in Millipore water (Millipore, Madrid, Spain). The pH of the solutions was adjusted by addition of HCl, NaOH or universal buffer of Theorell and Stenhagen [34] and the pH was measured in a Radiometer Copenhagen PHM240 pH/ion meter (Copenhagen, Denmark). UV-Vis absorption spectra were recorded in a Varian-Cary 100 Bio or 5000 spectrophotometer (Palo Alto, CA, USA). NMR experiments were run on a Bruker AMX 400 instrument (Billerica, MA, USA) operating at 400 MHz (^1^H) and 101 MHz (^13^C).

### 3.2. Flash Photolysis

Flash photolysis experiments were performed on a Varian Cary 5000 spectrophotometer with a Harrick fiber-mate attached to a 4-way cuvette holder (Ocean Optics, Dunedin, FL, USA) to perform light excitation perpendicular to the analyzing beam (sample compartment protected from daylight by black cardboard and black tape). As a pulsed white light source, a commercially available Achiever 630AF camera flash, placed in close contact with the sample holder, was used (time resolution of ca. 0.05 s) [13].

### 3.3. Synthesis

4′-Hydroxyacetophenone (1.41 g, 10 mmol) was treated with one equivalent of 2-[2-(2-chloroethoxy)ethoxy]ethanol, three equivalents of potassium carbonate and a catalytic amount of potassium iodide in 10 mL acetonitrile and stirred under reflux overnight. After cooling to room temperature, the solution was filtered and the filtrate was concentrated by rotary evaporation. The residue was treated with water and extracted with dichloromethane. The organic layer was dried over anhydrous sodium sulfate. After evaporation of the solvent, the product was purified by flash column chromatography on silica gel with gradient elution starting with ethyl acetate:hexane 1:1 and increasing the polarity to ethyl acetate:hexane 4:1. The product was isolated as uncolored oil in 60% yield (1.6 g). The identity of the desired 1-(4-[2-(2-(2-hydroxyethoxy)ethoxy)ethoxy]phenyl)ethanone by ^1^H-NMR spectroscopy. ^1^H-NMR (400 MHz, Chloroform-d) δ 7.9396 (d, *J* = 8.6 Hz, 2H), 6.9682 (d, *J* = 8.6 Hz, 2H), 4.2084 (d, *J* = 4.4 Hz, 2H), 3.8967 (t, *J* = 4.7 Hz, 2H), 3.7720–3.6876 (m, 6H), 3.6283 (t, *J* = 4.3 Hz, 2H), 2.5602 (s, 3H). 

A solution of 1-(4-[2-(2-(2-hydroxyethoxy)ethoxy)ethoxy]phenyl)ethanone (268 mg, 1 mmol) with one equivalent of 2,4-dihydroxybenzaldehyde (138 mg) in a mixture of glacial acetic acid/concentrated sulfuric acid (2 mL:0.5 mL) was stirred overnight at room temperature. Ethyl acetate was added to the red solution and a precipitate was formed. This precipitate was dissolved in methanol acidified with HCl and precipitated with diethyl ether. The dark orange solid was filtered, washed several times with diethyl ether and dried under vacuum. Yield: 120 mg, 28%. ^1^H-NMR (400 MHz, Methanol-*d*_4_ + DCl) δ 9.1597 (d, *J* = 8.7 Hz, 1H), 8.5104 (d, *J* = 8.7 Hz, 2H), 8.4002 (d, *J* = 8.7 Hz, 1H), 8.2044 (d, *J* = 8.9 Hz, 1H), 7.5690 (br, 1H), 7.4588 (dd, *J* = 8.9, 2.1 Hz, 1H), 7.3141 (d, *J* = 8.7 Hz, 2H), 4.3823 (t, *J* = 4.4 Hz, 2H), 3.9484 (t, *J* = 4.4 Hz, 2H), 3.7920–3.7343 (m, 2H), 3.7220–3.6604 (m, 6H), 3.5966 (t, *J* = 4.8 Hz, 2H). ^13^C-NMR (101 MHz, Methanol-*d*_4_ + DCl) δ 173.43, 170.60, 167.71, 160.56, 155.06, 134.09, 133.48, 122.72, 120.44, 117.59, 113.63, 103.72, 73.66, 71.82, 71.44, 70.45, 69.69, 62.20. Elemental analysis (%) calcd for C_21_H_23_O_6_. Cl. 1.5H_2_O (Mr = 433.88): C 58.13, H 6.04; Found: C 58.34, H 5.48.

## 4. Conclusions

The fact that anthocyanins follow the same multistate of chemical reactions of other flavylium derivatives, independent of whether they of a natural or synthetic origin, is a fortunate situation. The achievements from the study of these flavylium-based systems contribute to a better understanding of anthocyanins, which are the most significant compounds of the family. The interaction of flavylium compounds with cyclodextrins and cucurbiturils contributes to the understanding of the co-pigmentation phenomenon and may help in defining strategies to stabilize color in anthocyanins. Moreover, many synthetic flavylium compounds and also some natural ones, having the *trans*-chalcone as the major species at moderately acid solutions, are photochromic, allowing the obtainment of a panoply of color responses under light irradiation.

## Figures and Tables

**Table 1:** Parameters obtained from Figure 1 to Figure 2.

p*K′*_a_	p*K*_a_	*K*_h_*K*_t_*k*_i_ (M·s^−1^)	*K*_t_*k*_i_/*k*_−h_(M)	*k*_−i_ (s^−1^)	*k*_h_ (s^−1^)
2.85	4.2	3.6 × 10^−7^	2 × 10^−5^	2.7 × 10^−4^	0.018

**Table 2:** Equilibrium constants for compound **1**.

p*K′*_a_	p*K*_a_	*K*_h_ (M^−1^)	*K*_t_	*K*_i_
2.85	4.2	5.6 × 10^−7^	1.3	1.9 × 10^3^

**Table 3:** Rate constants for compound **1**.

*k*_h_ (s^−1^)	*k*_−h_ (M·s^−1^)	*k*_t_ (s^−1^)	*k*_−t_ (s^−1^)	*k*_i_ (s^−1^)	*k*_−i_ (s^−1^)
0.018	3.2 × 10^4^	0.57	0.45	0.5	2.7 × 10^−4^

**Table 4:** Equilibrium constants of the compound 4′,7-dihydroxy-3-methoxyflavylium.

p*K*_a_′	p*K*^^^_a_	p*K*_a_	*K*_h_/M^−1^	*K*_t_	*K*_i_
2.2	2.37	4.6	4 × 10^−3^	0.05	10.4

Estimated error 10%.

**Table 5:** Rate constants of the compound 4′,7-dihydroxy-3-methoxyflavylium.

*k*_h_ s^−1^	*k*_−h_ M^−1^·s^−1^	*k*_t_ s^−1^	*k*_−t_ s^−1^	*k*_i_ s^−1^	*k*_−i_ s^−1^
0.3	75	1.25 × 10^−2^	0.25	2.3 × 10^−4^	2.2 × 10^−5^

Estimated error 15%.

**Table 6:** Association constants (*K*) for the formation of host–guest complexes between flavylium cations and cucurbit[7]uril at pH 1. The estimated error is below 10%.

Flavylium	*K*/M^−1^
Compound **3**	2.1 × 10^6^
Compound **4**	5.1 × 10^5^
Compound **5**	5.5 × 10^5^
Compound **6**	2.7 × 10^5^
Compound **7**	5.1 × 10^6^
Compound **8**	8.0 × 10^6^
Compound **9**	5.0 × 10^6^

**Scheme A1:**
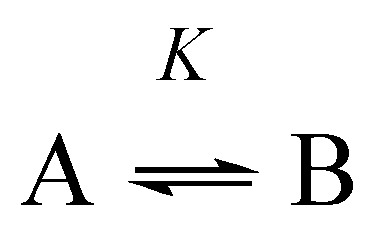
Equilibrium between A and B.

**Scheme A2:**
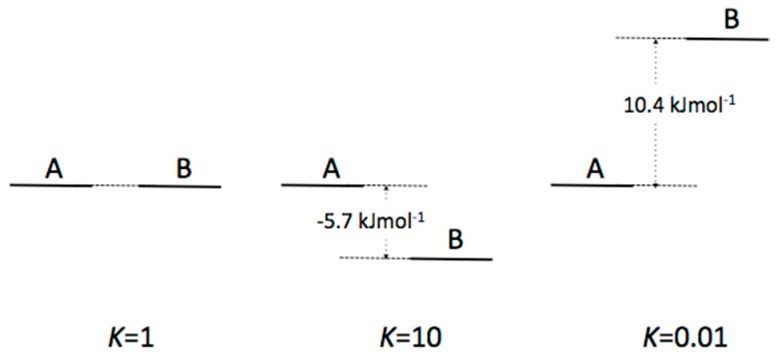
Energy level diagram for a hypothetical equilibrium between two species A (reference) and B.

**Scheme A3:**
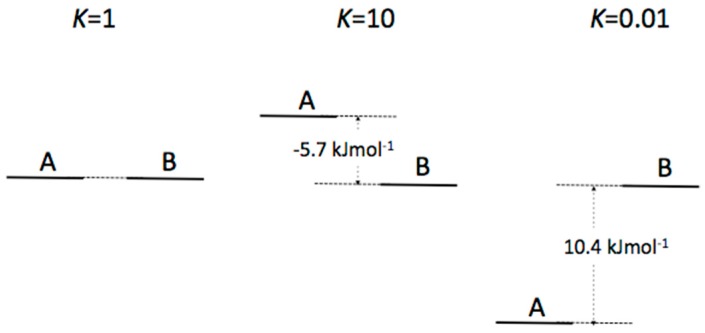
Energy level diagram for a hypothetical equilibrium between two species A and B (reference).

**Scheme A4:**
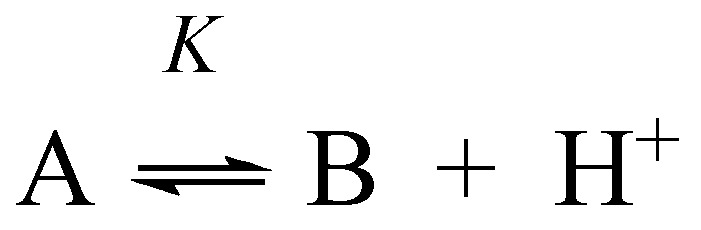
pH dependent equilibrium between A and B.

**Scheme A5:**
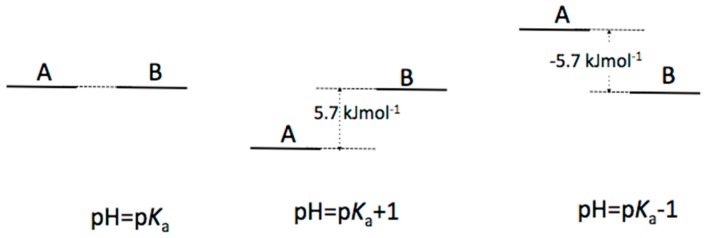
Energy level diagram for a hypothetical acid-base equilibrium between two species A and B.

**Scheme A6:**
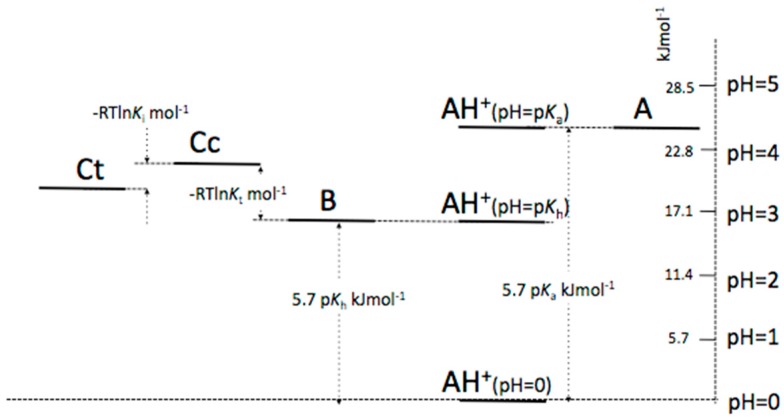
Energy level diagram for a hypothetical flavylium multistate.

**Figure B1:**
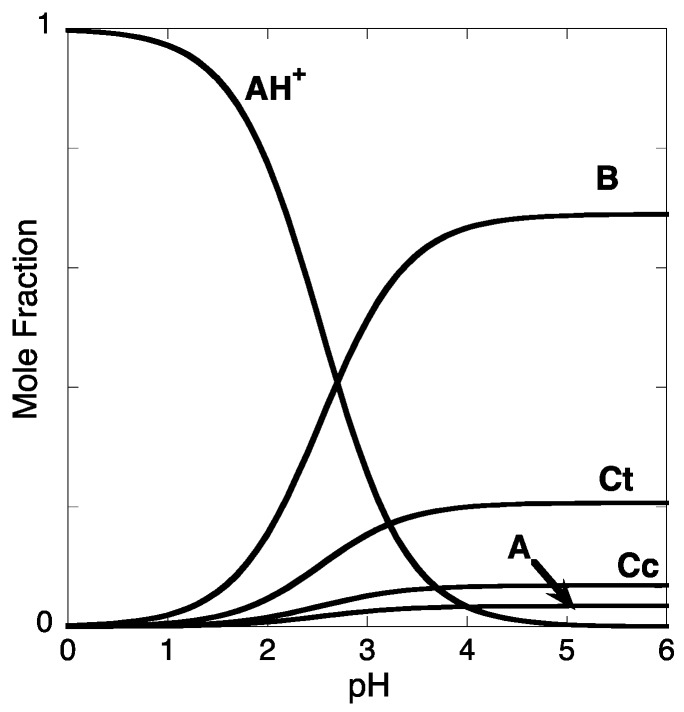
Mole fraction distribution for the following set of equilibrium constants *K*_a_ = 10^−4^ M; *K*_h_ = 2 × 10^−3^ M; *K_t_* = 0.1; *K*_i_ = 3 (*K*′_a_ = 2.9 × 10^−3^).

## Appendix A

Considering an equilibrium between two species A and B, Scheme A1, with equilibrium constant *K*: the standard Gibbs free energy ΔG° is defined by Equation (A1), where R is the gas constant T the absolute temperature (°K) [9].

(A1) $$\Delta G^{o}=-\mathrm{RTln}K$$

If the equilibrium constant is *K* = 1, ΔG° = 0, and the energy level of both species in Scheme A1 is equal. If the equilibrium constant is *K* = 10, ΔG° = −5.7 kJ·mol^−1^ at 25 °C and *K* = 0.01 leads to ΔG° = +11.4 kJ·mol^−1^ as illustrated in Scheme A2.

Due to the fact that the values of ΔG° are relative, it is more convenient for the next developments to maintain the energy level of B as the reference state, Scheme A3.

When the equilibrium is dependent on pH, as is the case of the proton transfer and hydration reactions in the flavylium network of chemical reactions, Scheme A4, a similar reasoning can be made considering that for each pH value there is an apparent equilibrium constant (which are different for different pH values).

(A2) $$K_{ap}=\frac{K}{\left[H^{+}\right]}=\frac{A}{\left[{\mathrm{AH}}^{+}\right]}$$

(A3) $$\Delta G^{o}=-\mathrm{RTln}\frac{K}{\left[H^{+}\right]}=-\mathrm{RTln}K-2.303\mathrm{RTpH}$$

When pH = p*K*_a_ ΔG° = 0 and by consequence

(A4) $$\mathrm{RTlnK}=-2.303\mathrm{RTp}K_{a}$$

and Equation (A3) can be rearranged to Equation (A5)

(A5) $$\Delta G^{o}=-2.303\mathrm{RT}\text{p}K_{a}-2.303\mathrm{RTpH}$$

According to Equation (A5), it is possible to calculate ΔG° for each pH value. In particular, if pH is one unit higher than p*K*_a_, ΔG° increases by 5.7 kJ·mol^−1^ and one unit lower decreased by the same amount, Scheme A5.

At this point, we have all the tools to apply these ideas to the flavylium network of chemical reactions. Taking pH = 0 as reference, ΔG° would be equal to −5.7p*K*_a_ kJ·mol^−1^. A scale in pH values can be constructed. In the case of the hemiketal (B) formation the procedure is identical. Final **Cc** can be positioned from **B** taking into account that ΔG° = −RTln*K*_t_ and **Ct** form **Cc** considering that ΔG° = −RTln*K*_i_, see Scheme A6.

## Appendix B

The following set of equations account for the different equilibria in which anthocyanins and related compounds are involved [9].

(B1) $${\mathrm{AH}}^{+}{+\;H}_{2}O⇌{A\;+\;H}_{3}O^{+}\;K_{a}$$

(B2) $${\mathrm{AH}}^{+}{+\;2H}_{2}O⇌{B\;+\;H}_{3}O^{+}\;K_{h}$$

(B3) $$B\;⇌\mathrm{Cc}\;K_{t}$$

(B4) $$\mathrm{Cc}\;⇌\mathrm{Ct}\;K_{t}$$

Considering now the equilibrium between **AH^+^** and the remaining “basic species”,

(B5) $${\mathrm{AH}}^{+}{+\;H}_{2}O⇌C{B\;+\;H}_{3}O^{+}\;K\prime_{a}$$

where [**CB**] = [**A**] + [**B**] + [**Cc**] + [**Ct**] (B6)

(B7) $$K\prime_{a}=\frac{\left\{\left[A\right]+\left[B\right]+\left[\mathrm{Cc}\right]+\left[\mathrm{Ct}\right]\right\}\left[H^{+}\right]}{\left[{\mathrm{AH}}^{+}\right]}$$

Using Equations (B1)–(B4)

(B8) $$K\prime_{a}=K_{a}+K_{h}+K_{h}K_{t}+K_{h}K_{t}K_{i}$$

The total concentration *C*_0_ can be written as in Equation (B9)

(B9) $$C_{0}=\left[{\mathrm{AH}}^{+}\right]+\left[A\right]+\left[B\right]+\left[\mathrm{Cc}\right]+\left[\mathrm{Ct}\right]=\left[{\mathrm{AH}}^{+}\right]\left(1+\frac{K_{a}}{\left[H^{+}\right]}+\frac{K_{h}}{\left[H^{+}\right]}+\frac{K_{h}K_{t}}{\left[H^{+}\right]}+\frac{K_{h}K_{t}K_{i}}{\left[H^{+}\right]}\right)$$

(B10) $$C_{0}=\left[{\mathrm{AH}}^{+}\right]\left(1+\frac{K\prime_{a}}{\left[H^{+}\right]}\right)$$

From Equation (B10), the mole fraction of **AH^+^** is obtained

(B11) $$\chi_{AH^{+}}=\frac{\left[{\mathrm{AH}}^{+}\right]}{C_{0}}=\frac{\left[H^{+}\right]}{\left[H^{+}\right]+K\prime_{a}}$$

Using the equilibrium constant of Equation (B1)

(B12) $$\chi_{A}=\frac{\left[A\right]}{C_{0}}=\frac{K_{a}\left[{\mathrm{AH}}^{+}\right]}{\left[H^{+}\right]C_{0}}=\frac{K_{a}}{\left[H^{+}\right]+K\prime_{a}}$$

by an identical procedure the mole fraction of the other species are also obtained.

(B13) $$\chi_{B}=\frac{K_{h}}{\left[H^{+}\right]+K\prime_{a}};\;\chi_{Cc}=\frac{K_{h}K_{t}}{\left[H^{+}\right]+K\prime_{a}};\;\chi_{Ct}=\frac{K_{h}K_{t}K_{i}}{\left[H^{+}\right]+K\prime_{a}}$$

The mole fraction distribution of several species as a function of pH can be calculated from Equations (B11)–(B13) (Figure B1). A simulation for *K*_a_ = 10^−4^ M; *K*_h_ = 2 × 10^−3^ M; *K_t_* = 0.1; *K*_i_ = 3 (*K*′_a_ = 2.9 × 10^−3^).
